# Association between antiretroviral therapy adherence and employment status: systematic review and meta-analysis

**DOI:** 10.2471/BLT.14.138149

**Published:** 2014-10-30

**Authors:** Jean B Nachega, Olalekan A Uthman, Karl Peltzer, Lindsey A Richardson, Edward J Mills, Kofi Amekudzi, Alice Ouédraogo

**Affiliations:** a Department of Epidemiology, University of Pittsburgh Graduate School of Public Health, 503 Parran Hall, 130 DeSoto Street, Pittsburgh, PA 15261, United States of America (USA).; b Warwick Centre for Applied Health Research and Delivery, University of Warwick, Coventry, England.; c Department of Psychology, University of Limpopo, Turfloop, South Africa.; d Department of Sociology, University of British Columbia, Vancouver, Canada.; e Stanford Prevention Research Center, Stanford University, Stanford, USA.; f HIV/AIDS and the World of Work Branch (ILOAIDS), International Labour Organization, Geneva, Switzerland.

## Abstract

**Objective:**

To assess the association between the employment status of human immunodeficiency virus (HIV)-infected individuals and adherence to antiretroviral therapy (ART).

**Methods:**

We searched the Medline, Embase and Cochrane Central Register of Controlled Trials databases for studies reporting ART adherence and employment status published between January 1980 and September 2014. Information from a wide range of other sources, including the grey literature, was also analysed. Two independent reviewers extracted data on treatment adherence and study characteristics. Study data on the association between being employed and adhering to ART were pooled using a random-effects model. Between-study heterogeneity and sources of bias were evaluated.

**Findings:**

The meta-analysis included 28 studies published between 1996 and 2014 that together involved 8743 HIV-infected individuals from 14 countries. The overall pooled odds ratio (OR) for the association between being employed and adhering to ART was 1.27 (95% confidence interval, CI: 1.04–1.55). The association was significant for studies from low-income countries (OR: 1.85, 95% CI: 1.58–2.18) and high-income countries (OR: 1.33, 95% CI: 1.02–1.74) but not middle-income countries (OR: 0.94, 95% CI: 0.62–1.42). In addition, studies published after 2011 and larger studies showed less association between employment and adherence than earlier and small studies, respectively.

**Conclusion:**

Employed HIV-infected individuals, particularly those in low- and high-income countries, were more likely to adhere to ART than unemployed individuals. Further research is needed on the mechanisms by which employment and ART adherence affect each other and on whether employment-creation interventions can positively influence ART adherence, HIV disease progression and quality of life.

## Résumé

**Objectif:**

Évaluer le lien entre le statut professionnel des personnes infectées par le virus de l'immunodéficience humaine (VIH) et le suivi de la thérapie antirétrovirale (TAR).

**Méthodes:**

Nous avons effectué des recherches dans les bases de données de Medline, d'Embase et du registre central de Cochrane des essais contrôlés (*Cochrane Central Register of Controlled Trials*) pour trouver les études relatives au suivi de la TAR et aux statuts professionnels qui ont été publiées entre janvier 1980 et septembre 2014. Des informations provenant d'un large éventail de sources alternatives, y compris la littérature grise, ont également été analysées. Deux examinateurs indépendants ont extrait les données sur le suivi du traitement et les caractéristiques de l'étude. Les données de l'étude sur le lien existant entre le fait d'avoir un emploi et le suivi de la TAR ont été regroupées en utilisant un modèle à effets aléatoires. L'hétérogénéité entre les études et les sources de biais ont été évaluées.

**Résultats:**

La méta-analyse a inclus 28 études publiées entre 1996 et 2014, qui, ensemble, ont impliqué 8743 personnes infectées par le VIH dans 14 pays. Le rapport des cotes (RC) regroupé global pour le lien entre le fait d'avoir un emploi et le suivi de la TAR était de 1,27 (intervalle de confiance à 95%, IC: 1,04–1,55). Ce lien était significatif pour les études dans les pays à revenu faible (RC: 1,85; IC 95%: 1,58–2,18) et dans les pays à revenu élevé (RC: 1,33; IC 95%: 1,02–1,74) mais il ne l'était pas dans les pays à revenu intermédiaire (RC: 0,94; IC 95%: 0,62–1,42). En outre, les études publiées après 2011 et les études plus vastes ont montré un lien moins fort entre le statut professionnel et le suivi thérapeutique que dans les études précédentes et les études plus restreintes, respectivement.

**Conclusion:**

Les personnes infectées par le VIH et ayant un emploi, en particulier dans les pays à revenu faible et les pays à revenu élevé, étaient plus susceptibles de suivre la TAR que les personnes sans emploi. Des recherches plus approfondies sont nécessaires pour étudier les mécanismes par lesquels le fait d'avoir un emploi et le fait de suivre la TAR s'affectent mutuellement et pour savoir si les interventions favorisant la création d'emplois peuvent influencer positivement le suivi de la TAR, la progression de la maladie due au VIH et la qualité de vie.

## Resumen

**Objetivo:**

Evaluar la relación entre la situación laboral de personas infectadas por el virus de la inmunodeficiencia humana (VIH) y la adherencia a la terapia antirretroviral (TAR).

**Métodos:**

Se realizaron búsquedas de estudios publicados entre enero de 1980 y septiembre de 2014 que informaran acerca de la adherencia a la TAR y la situación laboral en las bases de datos Medline, Embase y el Registro Cochrane Central de Ensayos Controlados. También se analizó la información de una amplia gama de fuentes, entre ellas, la literatura gris. Dos revisores independientes extrajeron los datos sobre la adherencia al tratamiento y las características del estudio. Los datos de los estudios sobre la asociación entre el empleo y la adherencia a la TAR se agruparon por medio de un modelo de efectos aleatorios y se evaluó la heterogeneidad entre los estudios y las fuentes de sesgo.

**Resultados:**

El metanálisis incluyó 28 estudios publicados entre 1996 y el 2014 que incluyeron 8743 individuos infectados por el VIH provenientes de 14 países. La razón de posibilidades agrupada general (OR) para la asociación entre estar empleado y la adherencia a la TAR fue 1,27 (intervalo de confianza del 95%, IC: 1,04–1,55). La asociación fue significativa en los estudios en países con ingresos bajos (OR: 1,85, IC del 95%: 1,58–2,18) ycon ingresos altos (OR: 1,33, IC del 95%: 1,02–1,74), pero no en los países con ingresos medios (OR: 0,94, IC del 95%: 0,62–1,42). Además, los estudios publicados después de 2011 y los de mayor tamaño mostraron una relación menor entre el empleo y la adherencia que, respectivamente, los estudios anteriores y más pequeños.

**Conclusión:**

Las personas infectadas por el VIH con un empleo, en particular en los países con ingresos bajos y altos, tenían más probabilidades de adherirse a la TAR que las personas desempleadas. Se necesitan investigaciones adicionales sobre los mecanismos por los que el empleo y la adherencia a la TAR se influyen mutuamente y sobre si las intervenciones de creación de empleo pueden influir positivamente la adherencia a la TAR, la progresión de la enfermedad del VIH y la calidad de vida.

## ملخص

**الغرض:**

تقييم الارتباط بين حالة التوظيف لدى الأشخاص المصابين بعدوى فيروس العوز المناعي البشري والالتزام بالعلاج بمضادات الفيروسات القهقرية.

**الطريقة:**

أجرينا بحثاً في قواعد بيانات Medline وEmbase وسجل كوكرين المركزي للتجارب الخاضعة للمراقبة من أجل معرفة الدراسات التي أبلغت عن الالتزام بالعلاج بمضادات الفيروسات القهقرية وحالة التوظيف وتم نشرها في الفترة من كانون الثاني/يناير 1980 إلى أيلول/سبتمبر 2014. وتم كذلك تحليل المعلومات المستمدة من نطاق واسع من المصادر الأخرى، بما في ذلك الأبحاث غير الرسمية. واستخلص مراجعان مستقلان البيانات الخاصة بالالتزام بالعلاج وخصائص الدراسة. وتم تجميع بيانات الدراسة المعنية بالارتباط بين التوظيف والالتزام بالعلاج بمضادات الفيروسات القهقرية باستخدام نموذج التأثيرات العشوائية. وتم تقدير التغايرية بين الدراسات ومصادر التحيز.

**النتائج:**

تضمن التحليل الوصفي 28 دراسة تم نشرها في الفترة من 1996 إلى 2014 والتي شملت معاً 8743 شخصاً مصاباً بعدوى فيروس العوز المناعي البشري من 14 بلداً. وكانت نسبة الاحتمال المجمعة للارتباط بين التوظيف والالتزام بالعلاج بمضادات الفيروسات القهقرية بشكل عام 1.27 (فاصل الثقة 95 %، فاصل الثقة: من 1.04 إلى 1.55). وكان الارتباط كبيراً بالنسبة للدراسات من البلدان المنخفضة الدخل (نسبة الاحتمال: 1.85، فاصل الثقة: 95 %، فاصل الثقة: من 1.58 إلى 2.18) والبلدان المرتفعة الدخل (نسبة الاحتمال: 1.33، فاصل الثقة: 95 %، فاصل الثقة: من 1.02 إلى 1.74) دون البلدان المتوسطة الدخل (نسبة الاحتمال: 0.94، فاصل الثقة: 95 %، فاصل الثقة: من 0.62 إلى 1.42). بالإضافة إلى ذلك، أوضحت الدراسات التي تم نشرها بعد عام 2011 والدراسات الأكبر نسبة ارتباط أقل بين التوظيف والالتزام عن الدراسات السابقة والصغيرة، على التوالي.

**الاستنتاج:**

يرجح التزام الأشخاص المصابين بعدوى فيروس العوز المناعي البشري الموظفين، لا سيما في البلدان المنخفضة والبلدان المرتفعة الدخل، بالعلاج بمضادات الفيروسات القهقرية عن الأشخاص العاطلين. ويتعين إجراء المزيد من الأبحاث بشأن الآليات التي يؤثر من خلالها التوظيف والالتزام بالعلاج بمضادات الفيروسات القهقرية كل منهما على الآخر وبشأن ما إذا كان بإمكان تدخلات إيجاد التوظيف أن تؤثر إيجابياً على الالتزام بالعلاج بمضادات الفيروسات القهقرية وعلى تفاقم مرض فيروس العوز المناعي البشري وعلى جودة الحياة.

## 摘要

**目的:**

评估艾滋病病毒（HIV）感染个人的就业状况和坚持抗逆转录病毒疗法（ART）之间的关联。

**方法:**

我们搜索了Medline、Embase和Cochrane对照试验中心注册数据库，寻找1980年1月至2014年9月发表的报告ART坚持和就业状况的研究。也对来自其他来源的广泛信息进行分析，其中包括灰色文献。由两名独立评审者抽取有关坚持治疗和研究特点的数据。使用随机效应模型汇聚有关受雇和坚持ART之间关联性的研究数据。评估了研究间异质性和偏差来源。

**结果:**

元分析包括了在1996年和2014年之间发布的28项研究，合计涉及来自14个国家的8743个HIV感染个人。受雇和坚持ART之间关联的总体汇总优势比（OR）为1.27（95%置信区间，CI：1.04–1.55）。来自低收入国家的研究的关联比较明显（OR： 1.85，95% CI：1.58–2.18），高收入国家也是如此（OR： 1.33，95% CI：1.02–1.74），但中等收入则不然（OR： 0.94，95% CI：0.62–1.42）。此外，在2011年之后发表的研究和更大的研究显示出的就业和坚持治疗之间的关联分别均比较早和较小研究更小。

**结论:**

较之失业的个人，就业的HIV感染个人（尤其在低收入和高收入国家）更可能坚持ART。需要进一步研究来了解就业和坚持ART相互影响的机制，以及创造就业干预是否对坚持ART、HIV疾病恶化和生活质量有积极的影响。

## Резюме

**Цель:**

Оценить связь между статусом занятости лиц, инфицированных вирусом иммунодефицита человека (ВИЧ), и соблюдением режима антиретровирусной терапии (АРТ).

**Методы:**

Был произведен поиск работ в базах данных Medline, Embase и в Кокрановском центральном реестре контролируемых клинических исследований, содержащих данные о соблюдении пациентами режима АРТ и их статусе занятости, которые были опубликованы в период с января 1980 по сентябрь 2014 года. Также была проанализирована информация из широкого круга других источников, в том числе, литературы, не индексированной в медицинских базах данных. Данные о соблюдении режима лечения и характеристиках исследованиий отбирались двумя независимыми экспертами. Данные исследованиий о связи между занятостью и соблюдением режима АРТ были объединены с использованием модели случайных эффектов. Была оценена гетерогенность между исследованиями и источники ошибок.

**Результаты:**

В мета-анализ было включено 28 исследований, опубликованных в период с 1996 по 2014 гг., которые в совокупности содержали данные по 8743 ВИЧ-инфицированным лицам из 14 стран. Суммарное обобщенное отношение шансов (ОШ) для связи между занятостью и соблюдением режима АРТ составило 1,27 (95%-ный доверительный интервал, ДИ: 1,04–1,55). Эта связь была существенной в исследованиях, выполненных в странах с низким уровнем дохода (ОШ: 1,85; 95%-ный ДИ: 1,58–2,18) и в странах с высоким уровнем дохода (ОШ: 1,33; 95%-ный ДИ: 1,02–1,74), но не в странах со средним уровнем дохода (ОШ: 0,94; 95%-ный ДИ: 0,62–1,42). Кроме того, исследования, опубликованные после 2011 года, и более крупные исследования показали меньшую связь между занятостью и соблюдением режима терапии по сравнению с более ранними и меньшими по масштабу исследованиями соответственно.

**Вывод:**

Имеющие трудовую занятость ВИЧ-инфицированные лица, особенно в странах с низким и высоким уровнем дохода, в большинстве своем, соблюдали режим АРТ, по сравнению с безработными лицами. Необходимы дальнейшие исследования механизмов взаимного влияния занятости на соблюдение режима АРТ, а также возможности положительного влияния мер по созданию занятости на соблюдение режима АРТ, прогрессирование ВИЧ-инфекции и качество жизни.

## Introduction

Recent data from the World Health Organization (WHO) and the Joint United Nations Programme on HIV/AIDS (UNAIDS) indicate that access to human immunodeficiency virus (HIV) treatment and care services in low- and middle-income countries has expanded dramatically.^1^ In 2012, over 9.7 million people living with HIV in these countries were receiving antiretroviral therapy (ART).^1^

Despite this increase, ensuring adherence to HIV treatment remains challenging in all countries. A meta-analysis of patients in North America (*n* = 17 573) and Africa (*n* = 12 116) estimated that only 55% and 77% in these areas, respectively, achieved over  80% adherence^2^ and a global meta-analysis, which included 33 199 patients on ART, reported that only 62% achieved over 90% adherence.^3^ Common barriers to adherence include medication side-effects, pill burden, the need to disclose HIV serostatus, a perception of feeling well, treatment fatigue and structural and psychosocial factors.^4^^,^^5^ For individuals, nonadherence can result in virological treatment failure, the development of drug resistance, disease progression and death.^6^^–^^9^ At the community level, nonadherence makes HIV transmission more likely,^10^^,^^11^ can substantially increase health-care costs, particularly for hospitalization to treat opportunistic infections,^12^ and can decrease productivity.^13^ One factor that has not been explored in depth is whether an individual’s employment status influences adherence to ART.^14^^,^^15^

Adherence to ART may be influenced by factors associated with the disease and its treatment, with the relationship between the patient and the health-care provider and with patients themselves, such as socioeconomic status which is often based on employment or occupational status in addition to educational level and income.^14^^,^^16^ Moreover, differences in adherence between people employed in the informal and the formal economy have been linked to gender roles and inequalities in employment status.^17^^,^^18^ A previous meta-analysis by our collaborative research group found that employed HIV-infected patients from low-, middle- and high-income countries were 39% more likely to adhere to ART than unemployed patients. However, the study included very few participants from middle- and low-income countries and therefore did not have sufficient statistical power to determine whether a country’s income level had a significant effect on the association between employment status and ART adherence.^19^

The aim of this systematic review and meta-analysis was to investigate the relationship between the employment status of HIV-infected individuals and ART adherence using updated data and a larger patient sample, which included more information from middle- and low-income countries.

## Methods

This meta-analysis was reported in accordance with the *Preferred reporting items for systematic reviews and meta-Analyses: the PRISMA statement*.^20^ Studies of any design were included if they satisfied the following criteria: (i) the study involved people living with HIV; (ii) participants were receiving highly active antiretroviral therapy; (iii) treatment adherence was assessed using objective or self-reported measures; and (iv) employment was considered a possible factor influencing adherence.

We searched the Medline, Embase and Cochrane Central Register of Controlled Trials databases for the period January 1980 to September 2014 (Box 1). In addition, we used a narrative literature review approach to analyse and summarize information on HIV treatment, particularly on adherence, from a range of sources including UNAIDS secretariat reports, scientific conference abstracts and other grey literature. We contacted individual researchers for details of unpublished studies.

Box 1 **Search terms used for studies on the association between employment status and adherence to antiretroviral therapy**

1. hiv infections/

2. HIV.ti.

3. human immunodeficiency virus.ti,ab.

4. HIV Infections/pc

5. HIV/ or HIV-1/

6. Acquired Immunodeficiency Syndrome/pc [Prevention & Control]

7. exp hiv/

8. exp hiv-1/

9. exp hiv-2/

10. Human immunodeficiency virus.mp.

11. hiv.mp.

12. or/ No. 1–11

13. blue collar.mp.

14. blue collar.ti,ab.

15. white collar.mp.

16. exp Social Class/

17. exp Adult/ or Occupations/

18. Agriculture/ec, ed, ma [Economics, Education, Manpower]

19. exp Employment/

20. job.mp.

21. exp work/

22. exp income/

23. manpower.mp.

24. socioeconomic.mp.

25. socio-economic.mp.

26. office.mp.

27. or/No. 13–26

28. exp Medication Adherence/

29. Adherence.mp.

30. Nonadherence.mp.

31. Compliance.mp.

32. or/ No. 28–31

33. No. 12 and 27 and 32

Two reviewers evaluated the eligibility of the studies identified and a third reviewer provided arbitration if there was a discrepancy. One reviewer extracted data, which were checked by others. The quality of the studies included was assessed using the Risk of Bias Assessment tool for Non-randomized Studies (RoBANS; details available from the corresponding author on request).^21^ The risk of bias in a study was graded as low, high or unclear on the basis of study features including the selection of participants (selection bias), consideration of confounding variables (selection bias), outcome measurement (detection bias), incomplete outcome data (attrition bias) and selective outcome reporting (reporting bias).

### Data extraction

For each study included, we recorded: the first author’s last name; the year of publication; the country where the study was performed; details of the study design; the years when data were collected; the type of controls in case–control studies; the duration of follow-up in cohort studies; sample size; details of exposure measures, such as indicators of occupation or employment; age; sex; the odds that an employed patient versus an unemployed patient would adhere to ART; and the variables controlled for. Study countries were classified by geographical area and categorized as low-, middle- or high-income, as defined by the World Bank for 2014.^22^ Study participants were defined as being of working age either by the study investigators or using the International Labour Organization’s standard definition.^23^ In addition, the International Labour Organization’s definition of employment was applied: Persons in employment comprise all persons above a specified age who during a specified brief period, either one week or one day, were in the following categories: paid employment and self-employment.^24^

Consequently, studies were considered eligible if their authors defined employed people as those who, during a specified brief period such as one week or one day: (i) performed some work for wage or salary in cash or in kind; (ii) had a formal attachment to a job but were temporarily not at work during the reference period; (iii) performed some work for profit or family gain in cash or in kind; or (iv) were with an enterprise such as a business, farm or service but who were temporarily not at work during the reference period for any specific reason.^24^

### Data synthesis

The meta-analysis was performed using the DerSimonian and Laird random-effects model to obtain a pooled estimate for the odds ratio (OR) and the associated 95% confidence interval (CI).^25^ The model was chosen since it takes into account both within- and between-study variability, as between-study heterogeneity was anticipated. Heterogeneity among studies was assessed by inspecting forest plots of the odds ratios from each study and by using the *χ*^2^ test for heterogeneity, with a 10% level of statistical significance, and the *I^2^* statistic, with which a value of 50% represented moderate heterogeneity. We used leave-one-study-out sensitivity analysis to evaluate the stability of the results and to test whether any one study had an excessive influence on the meta-analysis.^26^ In addition, we performed subgroup analyses to assess the influence of study design (i.e. cross-sectional versus prospective cohort), the country’s income group (i.e. low, middle or high), the adherence threshold and adherence measures. We used the *χ*^2^ test to subgroup differences and reported the *P*-value for interaction between pooled OR and study-level characteristics. We used a variance-weighted, least-squares regression approach to estimate the effect of the year of publication (i.e. trend analysis) and the study sample size on the association between employment status and ART adherence. For all tests, a probability less than 0.05 was considered significant. All statistical tests were two-sided. Analyses were performed using Stata version 12 (StataCorp. LP, College Station, United States of America).

## Results

**Fig. 1:**
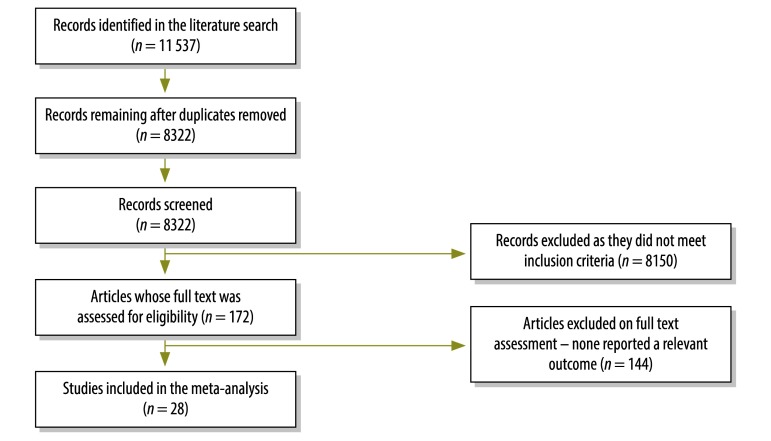
Flowchart showing the selection of studies for the meta-analysis of the association between employment status and adherence to antiretroviral therapy

A flow diagram of study selection is shown in Fig. 1. Twenty-eight studies, involving a total of 8743 patients from 14 countries, met criteria for inclusion in the systematic review (Table 1).^4^^,^^27^^–^^53^ The studies were carried out between 1996 and 2012 and publication took place between 1996 and 2014. Overall, 24 studies, involving 7484 of 8743 patients (86%), were cross-sectional, whereas the other four were prospective cohort studies. Eight studies, involving 1775 patients (20%), were carried out in the United States, three each were carried out in Ethiopia, India and South Africa and two were carried out in Uganda.

**Table 1:** Studies in the meta-analysis of the association between employment status and adherence to antiretroviral therapy, 14 countries, 1996–2014

First author of study	Year of publication	Study period	Study design	Study country	Country's income group^a^	Participants receiving ART at enrolment	Lower threshold for adherence to ART, %	Adherence measure	Sample size, *n*	Male participants, %	Age of participants,^b^ years	Unemployment, %	% ART adherence	Measure of association^c^
Singh^27^	1996	Not reported	Prospective cohort	United States	High	Yes	80	Pharmacy refill data	46	ND	23–68	41.0	63.0	Unadjusted
Singh^28^	1999	March 1996 to December 1997	Cross-sectional	United States	High	Yes	Not reported	Pharmacy refill data	123	ND	24–71	52.8	82.1	Unadjusted
Duong^29^	2001	Not reported	Cross-sectional	France	High	Yes	Not reported	Blood drug concentration	149	71.8	21–79	45.6	89.0	Unadjusted
Ickovics^30^	2002	Not reported	Cross-sectional from RCT	United States	High	Yes	95	Self-report questionnaire	93	88.0	19–61	23.9	63.0	Adjusted
Nachega^31^	2004	Not reported	Cross-sectional	South Africa	Middle	Yes	95	Self-report questionnaire	66	28.8	36.1 (10.1)	40.9	88.0	Unadjusted
Beyene^32^	2009	August to October 2009	Cross-sectional	Ethiopia	Low	Yes	95	Self-report questionnaire	422	43.6	32.2 (7.2)	59.0	93.1	Adjusted
Duggan^33^	2009	August to November 2006	Cross-sectional	United States	High	Yes	Not reported	Combination of methods	132	66.7	18–51	55.3	74.2	Unadjusted
Nakimuli-Mpungu^34^	2009	Not reported	Cross-sectional	Uganda	Low	Yes	90	Self-report questionnaire	122	21.3	36.0 (8.2)	34.4	82.8	Adjusted
Campos^35^	2010	May 2001 to May 2002	Prospective cohort	Brazil	Middle	Yes	95	Self-report questionnaire	293	65.9	ND	35.1	62.8	Adjusted
Giday^36^	2010	August to September 2008	Cross-sectional	Ethiopia	Low	Yes	95	Self-report questionnaire	510	38.6	15–63	39.6	88.2	Adjusted
Kunutsor^37^	2010	Not reported	Prospective cohort	Uganda	Low	Yes	95	Pharmacy refill data	392	35.2	32–45	55.4	93.1	Adjusted
Lal^38^	2010	2005	Cross-sectional	India	Middle	Yes	95	Self-report questionnaire	300	72.0	30–45	31.8	75.7	Unadjusted
Li^39^	2010	Not reported	Cross-sectional from RCT	Thailand	Middle	Yes	100	Self-report questionnaire	386	32.7	38.0 (6.4)	15.5	68.6	Adjusted
Peltzer^40^	2010	October 2007 to February 2008	Cross-sectional	South Africa	Middle	No	95	Self-report questionnaire	735	29.8	ND	59.6	82.9	Unadjusted
Sherr^41^	2010	2005 to 2006	Cross-sectional	United Kingdom	High	Yes	100	Self-report questionnaire	449	78.9	ND	42.8	42.8	Unadjusted
Venkatesh^42^	2010	January to April 2008	Cross-sectional	India	Middle	Yes	95	Self-report questionnaire	198	68.5	ND	21.9	49.0	Adjusted
Harris^43^	2011	June 2004 to December 2005	Cross-sectional	Dominican Republic	Middle	Yes	95	Self-report questionnaire	300	45.0	ND	53.0	76.0	Unadjusted
Juday^4^	2011	April to May 2007	Cross-sectional	United States	High	Yes	100	Self-report questionnaire	461	76.1	44.4 (9.3)	56.2	54.0	Adjusted
Kyser^44^	2011	March 2004 to June 2006	Prospective cohort	United States	High	Yes	100	Self-report questionnaire	528	78.0	20–66	41.0	84.0	Adjusted
Wakibi^45^	2011	November 2008 to April 2009	Cross-sectional	Kenya	Low	Yes	95	Self-report questionnaire	403	35.0	18–64	34.0	82.0	Unadjusted
King^46^	2012	February 2007 to December 2009	Cross-sectional from RCT	United States	High	Yes	100	Self-report questionnaire	326	72.1	45.9 (7.6)	79.0	60.4	Adjusted
Kitshoff^47^	2012	Not reported	Cross-sectional	South Africa	Middle	Yes	95	Pill count	146	27.4	31–42	65.0	68.0	Adjusted
Berhe^48^	2013	August 2012 to October 2012	Cross-sectional	Ethiopia	Low	Yes	95	Self-report questionnaire	174	46.0	38.5 (8.4)	20.1	40.8	Adjusted
Okoronkwo^49^	2013	Not reported	Cross-sectional	Nigeria	Middle	Yes	100	Self-report questionnaire	221	ND	ND	21.8	14.9	Unadjusted
Vissman^50^	2013	November 2008 to April 2009	Cross-sectional	United States	High	Yes	100	Self-report questionnaire	66	74.0	38.0 (10.3)	52.0	71.0	Unadjusted
Tran^51^	2013	2012	Cross-sectional	Viet Nam	Middle	Yes	95	Self-report questionnaire	1016	63.8	35.4 (7.0)	17.8	74.1	Adjusted
Saha^52^	2014	2011	Cross-sectional	India	Middle	Yes	100	Self-report questionnaire	370	58.4	33.5 (8.5)	33.0	87.6	Adjusted
Shigdel^53^	2014	2012	Cross-sectional	Nepal	Low	Yes	95	Self-report questionnaire	316	64.6	ND	22.5	86.7	Adjusted

ART: antiretroviral therapy; ND: not determined; RCT: randomized controlled trial.

^a^ Countries were categorized as low-, middle- or high-income, as defined by the World Bank for 2014.^22^

^b^ Participants’ ages are given as a range or as a mean (standard deviation).

^c^ Whether or not the measure of the association between employment status and adherence to antiretroviral treatment was adjusted for major confounding variables.

Twenty-seven studies, involving 8008 patients (92%), included participants who were already receiving ART. Twenty-three studies, involving 8171 patients (94%), defined adherence as receiving 95% or more of prescribed doses in a given period. In addition, 22 studies, involving 7755 patients (89%), used self-report questionnaires to assess adherence, whereas three used pharmacy refill data, one used the pill count, one used the blood drug concentration and one used a combination of methods. The median sample size was 300 participants (range: 46–1016). When reported, the percentage of males ranged from 21.3% to 88.0% between studies. The median percentage of unemployed participants was 41% (range: 16–79%) and the percentage of participants who adhered to ART ranged from 14.9% to 93.1%.

### Risk of bias

**Fig. 2:**
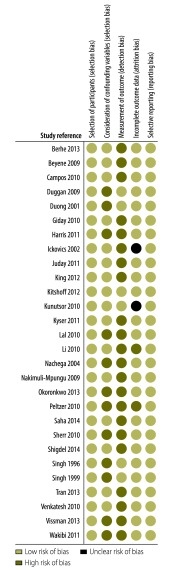
Risk of bias in studies in the meta-analysis of the association between employment status and adherence to antiretroviral therapy, 14 countries, 1996–2014

The results of our assessment of the risk of bias in all studies included in the meta-analysis are shown in Fig. 2. The risk of bias in the selection of participants was low in all studies. However, the risk of selection bias due to inadequate confirmation or consideration of confounding variables was low in 16 studies but high in 12: 16 studies adjusted for major confounding variables during the analysis phase, whereas the remaining 12 reported unadjusted associations between employment status and adherence to ART. The risk of detection bias due to inadequate outcome assessment was low in the six studies that assessed adherence using objective measures and high in the remaining 22, which used self-report questionnaires. The risk of attrition bias due to inadequate outcome data handling was low in 24 studies, unclear in two and high in the two studies in which more than 20% of patients were lost to follow-up. The risk of selective reporting bias was low in all studies.

### Association between adherence and employment

**Fig. 3:**
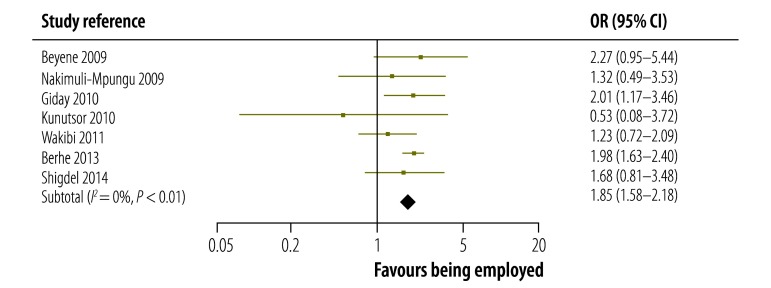
Association between being employed and adhering to antiretroviral therapy in studies from low-income countries, 2009–2014

CI: confidence interval; OR: odds ratio.

**Fig. 4:**
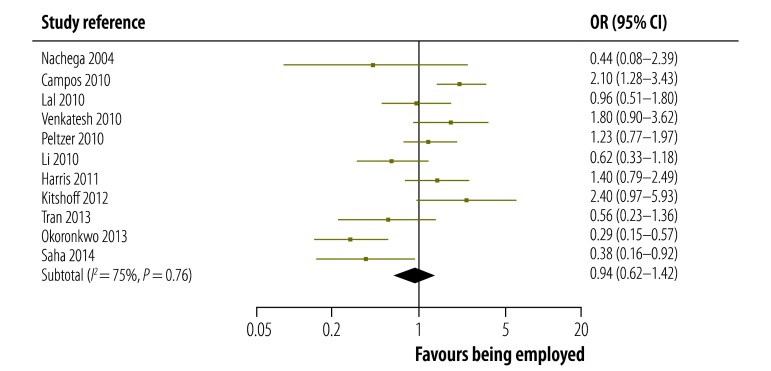
Association between being employed and adhering to antiretroviral therapy in studies from middle-income countries, 2004–2014

CI: confidence interval; OR: odds ratio.

**Fig. 5:**
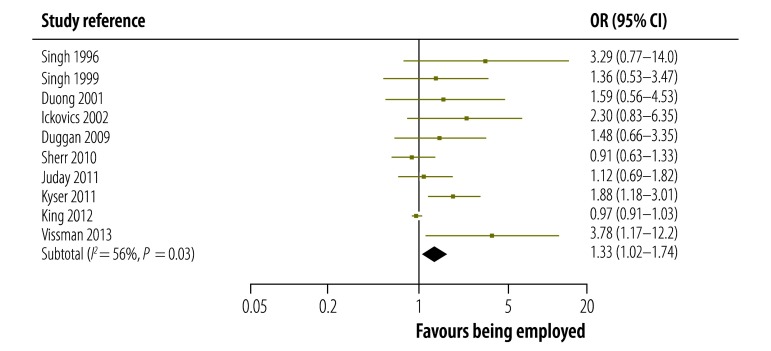
Association between being employed and adhering to antiretroviral therapy in studies from high-income countries, 1996–2013

CI: confidence interval; OR: odds ratio.

The strength of the association between being employed and adhering to ART reported in each study in low-, middle- and high-incomes countries is shown in Fig. 3, Fig. 4 and Fig. 5, respectively. The figures also give pooled estimates for the association in each income group. The pooled estimate for the OR in all countries was 1.27 (95% confidence interval, CI: 1.04–1.55). There was evidence of substantial statistical heterogeneity between the results of all studies (*I^2^*: 77%). The leave-one-study-out sensitivity analysis showed that no single study had an undue influence on the pooled estimate.

### Associations in subgroups

The magnitude and strength of the association between being employed and adhering to ART varied by country income group: it was highest in the seven studies from low-income countries, for which the pooled estimate for the OR was 1.85 (95% CI: 1.58–2.18). In the 11 studies from middle-income countries, the association was not significant as the pooled estimate for the OR was 0.94 (95% CI: 0.62–1.42). However, the association was significant in the 10 studies from high-income countries: the pooled estimate for the OR was 1.33 (95% CI: 1.02–1.74). In addition, the strength of the association was significantly higher in prospective cohort studies than in cross-sectional studies: the pooled OR was 2.05 (95% CI: 1.50–2.81) and 1.17 (95% CI: 0.95–1.44) for the two study types, respectively (*P*-value for interaction = 0.003). The association was significant in studies that used an adherence threshold less than 100% (OR: 1.59, 95% CI: 1.35–1.87) but not in those that used a threshold of 100% (OR: 0.90, 95% CI: 0.64–1.26; *P*-value for interaction = 0.003). We also found that the type of adherence measure used did not significantly influence the association between being employed and adhering to ART: the pooled OR was 1.21 (95% CI: 0.98–1.51) for studies that used self-report questionnaires compared with 1.67 (95% CI: 1.09–2.56) for those that used other measures (*P*-value for interaction = 0.19; Table 2).

**Table 2:** Association between being employed and adhering to antiretroviral therapy, by subgroup, 14 countries, 1996–2014

Subgroup	No. of studies	Pooled association		Subgroup heterogeneity,^a^ *P*
		OR (95% CI)	*I^2^*, %^b^	
**Income group^c^**				0.003
Low	7	1.85 (1.58–2.18)	0	NA
Middle	11	0.94 (0.62–1.42)	75	NA
High	10	1.33 (1.02–1.74)	56	NA
**Study design**				0.003
Cross-sectional	24	1.17 (0.95–1.44)	77	NA
Prospective cohort	4	2.05 (1.50–2.81)	0	NA
**Adherence threshold**				0.003
< 100%	20	1.59 (1.35–1.87)	17	NA
100%	8	0.90 (0.64–1.26)	78	NA
**Adherence measure**				0.19
Self-report questionnaire	22	1.21 (0.98–1.51)	81	NA
Other	6	1.67 (1.09–2.56)	0	NA

ART: antiretroviral therapy; CI: confidence interval; NA: not applicable; OR: odds ratio.

^a^ Subgroups were compared using the *χ^2^* test.

^b^ *I^2^* indicates the heterogeneity between the results of the studies in the subgroup.

^c^ Countries were categorized as low-, middle- or high-income, as defined by the World Bank for 2014.^22^

### Period and study size effects

**Fig. 6:**
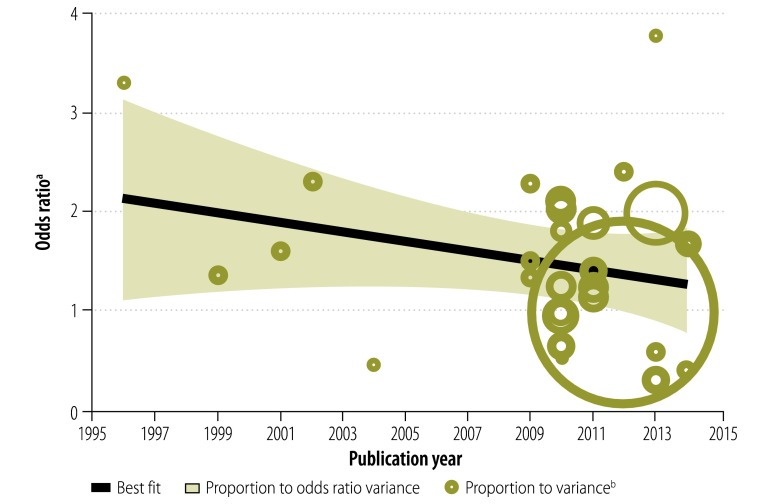
Association between being employed and adhering to antiretroviral therapy, by study publication year, 14 countries, 1996–2012

^a^ The odds ratio represents the odds that an employed patient would adhere to treatment compared with an unemployed patient.

^b^ The area of each circle is in inverse proportion to the precision of the odds ratio.

**Fig. 7:**
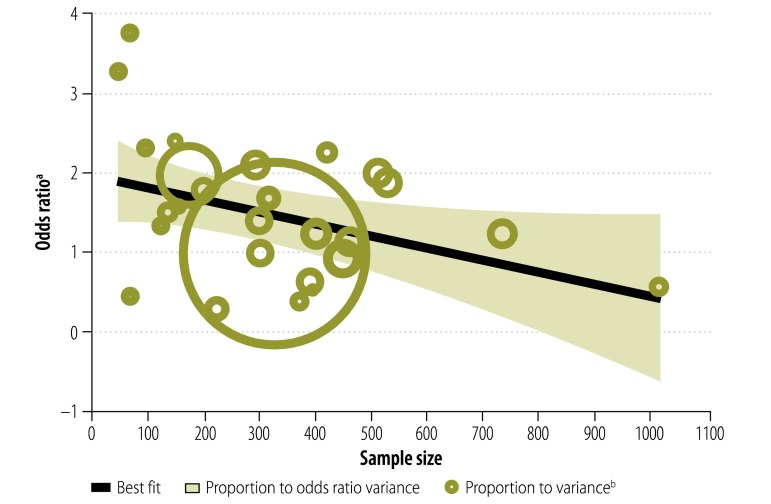
Association between being employed and adhering to antiretroviral therapy, by study sample size, 14 countries, 1996–2012

^a^ The odds ratio represents the odds that an employed patient would adhere to treatment compared with an unemployed patient.

^b^ The area of each circle is in inverse proportion to the precision of the odds ratio.

The association between being employed and adhering to ART was observed to change over time, i.e. there was a period effect. The magnitude of the association declined with the publication year – the pooled OR for studies published between 2011 and 2013 was lower than that for those reported before 2011 (Fig. 6). Similarly, we found that study size also significantly influenced the association, which was more pronounced in those with small samples (Fig. 7). For each additional 100 study participants, the magnitude of the association decreased by 9% (*P* = 0.001).

## Discussion

Overall, we found that patients with HIV infections who were employed were 27% more likely to adhere to ART than those who were unemployed. This is in agreement with the results of our previous meta-analysis^19^ and of others^5^ who reported that one of the barriers to ART adherence in both developed and developing countries was financial constraints, which may be considered a proxy for unemployment. One difference from our previous report is that the magnitude and strength of the association between being employed and adhering to ART were highest in studies from low-income countries. Hence, unemployment may have a greater effect on ART adherence in these settings.

It is possible that employment facilitates adherence to HIV treatment because it is associated with, for example, increased social support, better structuring of time and improved psychosocial well-being – these associations have all been documented in the general population.^54^^,^^55^ A review of 16 longitudinal studies showed evidence that unemployment has a negative effect on mental health^56^ and other studies have found that depression and impaired psychosocial well-being are associated with poor ART adherence.^57^ Employment may also promote increased material well-being, for example, by improving food security and housing quality and by reducing poverty – all three are known to be associated with adherence to HIV treatment.^5^^,^^58^^,^^59^ In addition, employment may promote adherence to ART by improving access to medical services through employer-sponsored health programmes that specifically encourage HIV treatment and by having a positive effect on physical and mental health-related quality of life.^5^^,^^60^ Conversely, the association may, in part, be due to the positive effect of ART on an individual’s ability to find and retain employment, i.e. reverse causation. For example, it was found that HIV-infected workers who were not receiving ART were almost twice as likely to report being unable to work in the previous week than those who recently began and were adherent to ART.^61^ Another study on more than 2000 HIV-infected adults enrolled in an ART programme estimated that four years after ART initiation, employment had returned to about 90% of the rates observed in the same patients three to five years before ART initiation.^62^ Further prospective, longitudinal, cohort studies of employment status, ART adherence and other factors could help tease out the relative effects of ART adherence and employment on each other.

Despite mutual reinforcement between employment and ART adherence, it can be difficult for individuals to maintain adherence. For example, it was found that the main reasons for the discontinuation of antiretroviral therapy in the workplace in South Africa were: individuals being uncertain about their own HIV status and about the value of ART; poor relationships between patients and health-care providers; and discrimination in the workplace.^63^ Furthermore, workplace participants also felt that the follow-up visits required by ART clinics created problems for them with their employers. The most frequently cited reason for treatment discontinuation among these individuals was harassment and discrimination by line managers who refused to grant time off from work for clinic attendance. For public sector employees, the main reasons included relocation away from HIV care providers and having insufficient money for transportation to clinical facilities.^63^ These findings suggest there is a considerable need to promote awareness of the importance of ART adherence and to develop employment arrangements among both employers and employees that encourage adherence.

### Adherence, employment and gender

Unexpectedly, we found no evidence that gender had a significant influence on the association between employment status and ART adherence. Several factors could account for this finding. There is a gender gap in access to antiretroviral therapy. In most areas of the world, and especially in settings with a high burden of HIV infection, women are more likely than men to access both ART and supportive HIV services, such as targeted counselling and programmes for the prevention of mother-to-child HIV transmission offered by antenatal services.^64^ Conversely, men may have better access to employment than women and employment could facilitate increased adherence among men.^65^^,^^66^

### Period effect

Our finding that the association between being employed and adhering to ART declined over time may reflect changes in ART regimens, such as the trend towards once-daily dosing, reductions in the pill burden and side-effects,^67^^,^^68^ better access to treatment for both employed and unemployed individuals or a drop in the cost of HIV medications.^1^ Further research is needed to determine whether this finding is specific to the studies we included or whether it signifies a true decrease in the association over time.

### Limitations

The findings of this meta-analysis should be interpreted with caution. The observational nature of the data – 85% of the studies included had a cross-sectional design – limited our ability to draw causal inferences and our findings may be affected by reverse causation bias or by other unknown confounding factors that were not adjusted for. Additionally, we found significant heterogeneity across the studies, which suggests that a substantial percentage of the variability in effect estimates was due to heterogeneity rather than to sampling errors, i.e. to chance. Much of the heterogeneity observed may be explained by differences in the adherence threshold, study sample size and study design. Nevertheless, even when there is substantial heterogeneity, meta-analysis is regarded as preferable for data synthesis to qualitative or narrative interpretation since these approaches can lead to misleading conclusions that should not be generalized beyond the scope of the analysis.^69^ The ability to draw conclusions from quantitative data is an important feature of meta-analyses and its absence is one reason for avoiding narrative interpretations without a data synthesis. It is worth noting that the heterogeneity observed in the current study appears to be the norm rather than the exception in meta-analyses of ART adherence.^14^^,^^15^ An additional potential limitation is that none of the studies included compared treatment adherence across different types of occupation. Further, we found evidence for a small study effect and it is possible that the observed magnitude of the association between being employed and adhering to ART could have been inflated by studies with small sample sizes.

Despite these limitations, our study had important strengths. We conducted comprehensive searches of databases to ensure that all relevant, published studies were identified. We also carried out meta-regression analyses to determine whether any particular study-level factor explained the results or could account for the observed variations between studies. In performing this comprehensive and robust review of the existing literature, we identified gaps in the current literature on determinants of ART adherence.

### Areas for future research

Our review was unable to address all pertinent questions on the association between employment status and optimal adherence to HIV treatment. Our study findings indicate a need for a range of further research: (i) to investigate mechanisms by which employment and ART adherence affect each other; (ii) to determine how different types of employment can differentially influence ART adherence; (iii) to study the association and interaction between employment and adherence to medications for chronic comorbid diseases, e.g. hypertension, diabetes and asthma in HIV-infected or uninfected individuals; (iv) to conduct interventional studies of how employment-creation programmes can positively influence HIV treatment adherence, disease progression and quality of life; and (v) to carry out cost-benefit and cost-effectiveness analyses of selected employment-creation interventions for people living with HIV.
